# Change in Sunflower Oil Quality and Safety Depending on Number of Deodorisation Cycles Used

**DOI:** 10.3390/foods13162555

**Published:** 2024-08-16

**Authors:** Mariia Andreevna Makarenko, Alexey Dmitrievich Malinkin, Vladimir Vladimirovich Bessonov

**Affiliations:** Food Chemistry Laboratory, Federal Research Centre of Nutrition and Biotechnology, 109240 Moscow, Russia; malinkin@ion.ru (A.D.M.); bessonov@ion.ru (V.V.B.)

**Keywords:** repeated deodorisation, sunflower oil, oxidation stability, volatile oxidation products, hexanal, 3-monochloropropane-1,2-diol fatty acid esters, glycidyl fatty acid esters, fatty acids

## Abstract

Deodorisation remains a beneficial aspect of the processing of edible oils and fats and is required during the first refining and after transportation, storage, and/or further processing, such as interesterification. While there is awareness among the scientific community that repeated deodorisation may negatively impact product quality, according to some technical and processing requirements, oils, fats, and their blends can still be treated with up to 3–4 cycles of deodorisation. However, the precise changes caused by sequential deodorising processes remain unknown. This study analysed fatty acid compositions, peroxide values, anisidine values, volatile profiles, and monochloropropanediol (MCPDEs) and glycidyl (GEs) fatty acid ester contents in pressed and repeatedly deodorised sunflower oils (SFOs). The latter had higher levels of saturated fatty acids (SFAs); monounsaturated fatty acids (MUFAs); and trans fatty acids (TFAs); as well as volatile aldehydes, such as pentanal, hexanal, (E)-2-hexenal, and (E)-2-heptenal, and MCPDE contents with each successive deodorisation. Most of these compounds have the potential to cause harmful health effects. Therefore, it is necessary to limit the number of edible oil deodorisation cycles in order to maintain their quality and safety.

## 1. Introduction

Edible oils and fats are important parts of the human diet. In today’s market, there are two main types of oils and fats available: crude (or pressed) and refined. Crude oils undergo little or no processing, while refined oils undergo chemical and/or physical processing. Pressed oils and fats are generally considered healthier, but refined oils have a broader range of applications since they lack specific tastes and flavours and are cheaper [1]. Refining, particularly deodorisation, is considered to increase the shelf life of crude edible oils and fats [2].

Unfortunately, the term “refined” on the label does not indicate that oil has undergone a single refining process. Partial refining is officially required by FEDIOL (the federation representing the European Vegetable Oil and Proteinmeal Industry in Europe) as an intermediate processing step after transportation in bulk in various countries, including the European Union [3,4] and the Russian Federation [5]. The requirement applies to oils and fats, including those that have undergone prior refining and are not intended for direct consumption but rather for subsequent use in production. This intermediate processing should involve at least deodorisation [3,6], which is intended to decrease the impact of oxidation and enhance the quality and safety attributes of oils [7] and/or to mitigate technical contamination to meet regulatory requirements. The FEDIOL requirement is mandatory for producers worldwide who use tanker traffic and is additionally employed in both road and rail tank transportation.

Additional processing is a regular procedure for fat blends, such as spreads, shortenings, and margarines, which are usually interesterified mixtures of two or more fats or oils that have been previously refined and deodorised at least once because interesterification also requires deodorisation to improve the quality of products [8]. Prolonged product storage requires additional deodorisation as well. Thus, in some cases, oils, fats, or products thereof may undergo up to three or more deodorising cycles [6].

At the same time, excess processing, particularly additional deodorisation, is not recommended by the Codex Alimentarius commission due to potential adverse effects on product quality [7]. Several methods have been developed to modify the deodorising step, with the aim of saving labile compounds like fatty acids [9] and sterols [10], as well as reducing compounds responsible for unpleasant or foreign smells, indicators of rancid taste [11,12,13], and certain harmful substances. These methods target particularly volatile second-order oxidation products [6], as well as peroxide or acid values and contamination by MCPDE and GE [6]. 3-MCPD, a hydrolysis product of 3-monochloropropanediol fatty acid esters, is classified by the IARC as group 2B (“possibly carcinogenic to humans”), and glycidol, a product of glycidyl fatty acid esters, is classified as group 2A (“probably carcinogenic to humans”). All these esters, including 2-monochloropropanediol, are capable of extensive hydrolysis to their corresponding free forms following oral administration [14,15,16]. However, an increase in tumour incidence in vivo was observed only for free glycidol, with less significant increases observed for glycidyl esters. The main effect of 3-MCPD exposure is nephrotoxicity, which was observed in rats [17]. Data on 2-MCPD toxicity are still considered to be insufficient to undertake a risk characterisation. However, the overall renal effects of 2-MCPD appear to be less harmful than those of 3-MCPD [16]. 

At the same time, modifying or introducing new technology can be costly and time-consuming, particularly for small- and medium-sized manufacturers.

Up to now, repetitive heating has mainly been studied in connection with frying oils and their stability [18,19] because frying is one of the severest treatments to which fats and oils can be subjected and is a widely used cooking technique [19]. Frying oils and fats that are specially formulated to withstand repeated heating and cooling cycles are different from non-specialised oils and fats, which are likely to be poorly suited to such conditions. However, information regarding the quality and safety parameters of repeatedly deodorised edible fats and oils is lacking. Thus, the present work aimed to determine the possible effects of repeated deodorisation on the chemical composition, safety, and oxidative stability of edible oils at the earliest stages of oxidation.

## 2. Materials and Methods

All reagents used were of analytical grade.

### 2.1. Samples and Their Refining and Deodorisation Conditions

All sunflower oils (SFOs), fresh and deodorised, were provided by a local oil production plant. At our special request, the plant used their own pilot refining facility and sunflower seeds in order to replicate real plant conditions and processes, from extraction to packaging, instead of using a model of the full technological process simulated in the laboratory. All information regarding subsequent refining processes was provided by the aforementioned oil production plant.

At the plant, SFO was extracted from seeds by pressing at 75–110 °C. Immediately after pressing, the oil samples were refined in the following way: the pressed oil was conditioned with 30% *w*/*w* lemon acid at 70–75 °C, then neutralised with 12–13% *w*/*w* NaOH at 70–75 °C and washed with water at 90–95 °C. The next step was bleaching, which was performed at 90 ± 5 °C for 75 ± 5 min. In this step, the sunflower oil was treated again with 30% lemon acid and then with bleaching clay (3 g/kg oil). Then, the SFO was deodorised repeatedly from one to four times with the same oil at 220 ± 2 °C for 75 ± 5 min. Immediately after pressing and each deodorisation cycle, samples were collected, packed into foil bags with a lock and a tamper-evident seal, and sent to the Food Chemistry Laboratory of the Federal Research Centre of Nutrition and Biotechnology using a portable freezer. Prior to packing, all the samples were measured for PV value as a routine quality measurement indicator during processing in the plant’s own laboratory.

Thus, we were provided with one sample of sunflower oil after a different number of refining and deodorising cycles treated in plant conditions (Table 1). All the samples were stored at −25 °C prior to analyses.

### 2.2. Fatty Acids Composition and Content

The samples were analysed in duplicate. Approximately 10 mg of each sample was weighed in vials with PTFE-lined caps. Then, 850 μL of undecanoic acid methyl ester (Supelco, Bellefonte, PA, USA), C = 0.593 mg/mL; glyceryl tritridecanoate (TRC, Toronto, ON, Canada), C = 0.513 mg/mL; and butylhydroxytoluene (as antioxidant) (SAFC P.O., St. Louis, MO, USA), C = 0.012 mg/mL (Supelco, Bellefonte, PA, USA) mixture solution in methanol (EKOS-1, Moscow region, Russian Federation) were added. Next, 1 more ml of methanol, 20 µL of hexane (EKOS-1, Moscow region, Russian Federation), and 20 µL of acetyl chloride (Acros organics, Pittsburgh, PA, USA) were added to the samples. The tubes were tightly sealed, shaken intensively, and then heated for 1 h at 80 °C for methylation. Following cooling to room temperature, 2.5 mL of hexane and 100 µL of Milli-Q grade water (Milli-Q Academic, Merck KGaA, Darmstadt, Germany) were added, and the samples were stirred vigorously for about 10 s using a laboratory shaker (IKA-Werke GmbH & Co. KG, Staufen, Germany). For each sample, one mL of the upper layer containing the fatty acid methyl esters was transferred to a GC-FID vial for analysis.

Conditions for GC-FID analysis were as follows: sample injection volume—1 μL; split mode, 30:1; carrier gas, nitrogen; flow rate, 0.9 mL/min. The injector temperature was set at 260 °C; the detector temperature was set at 240 °C. The separation conditions were as follows: initial temperature was 140 °C (isotherm for 5 min.), then increasing at 4 °C/min up to 220 °C (isotherm 25 min). The data were collected and processed using Agilent ChemStation Rev.B.04.03 [16] and Microsoft® Office Excel® 2007 software (12.0.6787.5000). The content of fatty acids was calculated using undecanoic acid methyl ester as the internal standard. The completeness of the interesterification and extraction of methyl esters into hexane was verified using glyceryl tritridecanoate. Conversion factors for the methyl esters to free forms were used to calculate total FA content, as previously described by [20].

A 7890A chromatograph, equipped with an autosampler 7683B Series (Agilent Technologies, Santa Clara, CA, USA), an FID detector, and a column Select FAME 100 m length, 25 mm internal diameter × 0.25 µm film thickness (Agilent Technologies, Santa Clara, CA, USA) were used for the measurement. In order to identify the fatty acids, a FAME 37 Component Mix in dichloromethane (Supelco, Bellefonte, PA, USA) and a Linoleic Acid Methyl Ester Mix cis/trans isomers in dichloromethane (Sigma-Aldrich, WY, USA) were employed.

### 2.3. Peroxide Value Measurement (PV)

The determination of PV was conducted in duplicate according to the standard procedure [21], with slight modifications. Briefly, approximately 4 g of each sample was transferred and weighted in 100 mL conical flasks. Then, 20 mL of an acetic acid/chloroform (EKOS-1, Moscow region, Russian Federation) mixture in a 2:1 *v*/*v* was added to the flasks, which were then agitated. Next, 1 mL of a 50% KI (Vekton, St. Petersburg, Russian Federation) solution was added, and the flasks were immediately transferred to a dark location. Following a 20-minute reaction period, the samples were diluted with 50 mL Milli-Q water and subjected to titration with a 0.01 N Na_2_S_2_O_3_ solution (Vekton, St. Petersburg, Russian Federation). The concentration of the Na_2_S_2_O_3_ titrant was determined before the analysis according to [22]. The endpoint was determined visually by solution’s colour change following the addition of a 1% potato starch solution (Chimmed Group, Moscow region, Russian Federation) near the end of the titration.

### 2.4. Anisidine Value (AV)

The AV measurement was conducted according to [23]. The samples were analysed in triplicate.

### 2.5. Volatile Profile Analysis

Headspace solid-phase microextraction (HS-SPME) coupled to gas chromatography and flame ionisation and mass spectrometry detection (GC-FID/MS) was employed for the analysis of volatiles in SFO samples. FID data were used to obtain areas of analytes, thereby facilitating more accurate comparisons between samples. MS data were used for identification purposes. During this work, we focused on identifying specific scent footprints and a comparative assessment of volatile profiles rather than absolute quantitification of volatiles.

#### 2.5.1. Extraction of SFO’s Volatiles by HS-SPME

Following defrosting at room temperature, 10 mL of each sample was transferred into a 20 mL vial with a magnetic stirrer and sealed with a crimp hole cap and PTFE beige septa (Machery-Nagel, Duren, Germany). The samples were then incubated in a preheated drying oven (Binder, Tuttlingen, Germany) at 40 °C for 15 min. Then, the samples were transferred into a glass vessel containing water that had been preheated to 40 °C. The glass vessel was placed on a hotplate magnetic stirrer, and a thermometer was used to control the temperature of the water. Then, the 50/30 μm DVB/CAR/PDMS fibre (Supelco, Bellefonte, PA, USA) was positioned over the, ensuring that a minimum of 5 mm was maintained between the fibre and the sample surface. The fibre was incubated over the sample for 20 min, after which it was rapidly removed from the headspace and immediately placed into the previously heated GC injector (255 °C, splitless mode) for 3 min to desorb volatiles.

Before analysis, the fibre was conditioned in accordance with the manufacturer’s recommendations. Also, blank analyses were conducted periodically under the conditions described below and without samples in order to control the presence of any volatile compounds that may have been adsorbed on the fibre from the laboratory air.

#### 2.5.2. GC–FID and GC–MS Analysis

Volatiles were analysed using the 7890A GC instrument, coupled to both the FID and the quadrupole mass detector (MSD) 5975C (Agilent Technologies, Santa Clara, CA, USA). The volatile compounds were separated on a Supelcowax 10 column (bonded polyethylene glycol, 60 m, 0.53 mm i.d. × 1.0 μm film thickness) (Supelco, Bellefonte, PA, USA), with the following temperature program: the temperature was maintained at 35 °C for 5 min, then increased at a rate of 4 °C/min to 220 °C and held for 40 min. A Deans switch was positioned after the column to divide the mobile phase, with one portion directed to the FID and the other to the MSD. The FID temperature was set to 250 °C; carrier gas was helium (2.8 mL/min). The MS scans were performed in a TIC operation mode, with the following parameters: transfer line temperature of 260 °C, ion source and quadrupole temperatures of 230 °C and 150 °C, respectively, and an acquisition mass range of 35–400 *m*/*z*. All mass spectra were acquired in electron-impact (EI) mode with an ionisation voltage of 70 eV.

#### 2.5.3. Identification of Volatiles

The MS spectra of each peak with a height exceeding 3 baseline standard deviations were compared to the corresponding MS spectra available in the NIST Mass Spectral Search Program for the NIST/EPA/NIH Mass Spectral Library, version 2.0 g (built May 19 2011). Matching factor with values exceeding 700 was taken as an initial identification criterion. The second criterion was the Kovats indices, which were calculated using a C5-C24 n-alkanes series (ChromLab, Moscow region, Russian Federation) and then compared to the available Kovats indices for polar columns at the PubChem [24] and the NIST Chemistry WebBook [25] resources. Additionally, the Good Scents Company Information System was used to match identified volatiles and their aroma profile [26].

### 2.6. MCPD Esters and Glycidyl Esters Measurement

Two distinct batches of oils were subjected to analysis, with each batch analysed in triplicate. The measurement was conducted according to the previously developed procedure, which is based on a long-term alkaline transesterification and GC-MS/MS determination [27]. Briefly, 100 ± 20 mg of the sample was weighted into both A and B flacons. To the A flacon, 50 μL of 3-MCPD-d5 and 50 μL of 3-MBPD-d5 solutions (10 μg/mL each) in methanol were added. To the B flacon, 100 μL of the 1,2-PP-3-MCPD-d5 and 1,3-PP-2-MCPD-d5 combined mixture in toluene was added (5 μg/mL of each as free 2- and 3- MCPD) (all standards TRC, Toronto, ON, Canada). Both flacons were then poured with 600 μL of diethyl ester (Chimmed Group, Moscow region, Russian Federation), vortexed, and placed in a freezer at −25 °C. Following a 16-hour period, 500 μL of 2.5 mg/mL NaOH (Chimmed Group, Moscow region, Russian Federation) in methanol was added, vortexed, and then samples were placed back in the freezer. Following a 16-hour period, 600 μL of the acidified 55% NaBr (Chimmed Group, Moscow region, Russian Federation) solution in water was added, and the flacons were immediately vortexed. The upper layer was then evaporated in a vacuum using a centrifugal microconcentrator (Eppendorf, Hamburg, Germany). Subsequently, 600 μL of hexane was added, and the samples were vortexed and left to stand until two phases were observed. The upper phase was then removed. The procedure was repeated twice. Subsequently, 900 μL of diethyl ester and ethyl acetate (Chimmed Group, Moscow region, Russian Federation) solution in a 2:3 *v:v* ratio was added to samples, which were then vortexed and left to stand until two phases were observed. The upper phase was then transferred to a new flacon. The procedure was repeated twice. The combined extracts were evaporated in vacuum using a centrifugal microconcentrator. After that, 1 mL of 20 mg/mL of phenyl boronic acid (TRC, Toronto, ON, Canada) solution in diethyl ester was added. Following a 15-minute reaction period, the diethyl ester was evaporated in the centrifugal microconcentrator, and 1 mL of isooctane (Chimmed Group, Moscow region, Russian Federation) was added. The samples were vortexed and subjected to centrifugation, and 200 μL of a transparent solution was transferred to GC vials.

The injection and detection conditions were as follows: sample injection volume, 1 μL; splitless mode; carrier gas, helium; flow rate, 1.2 mL/min. The interphase temperature was set at 280 °C, the source temperature was set at 230 °C, and the quadrupole temperature was set at 150 °C. Triple quadrupole was operated in MRM mode. The separation conditions were as follows: initial temperature was 60 °C, after which it increased at a rate of 5 °C/min up to 190 °C, then increased at a rate of 20 °C/min up to 280 °C (isotherm for 5 min), and finally, it increased again up to 300 °C (isotherm for 5 min).

A quantitative analysis was performed using calibration curves prepared with standard solutions of 3-MCPD, glycidol, 1,2-PP-3-MCPD (as free 3-MCPD), and 1,3-PP-2-MCPD (as free 2-MCPD), with concentrations of 0.01 μg/mL, 0.05 μg/mL, 0.2 μg/mL, 0.50 μg/mL, 2.00 μg/mL, and 5.00 μg/mL for each compound. The curves were acceptable if the coefficient of determination R^2^ was greater than 0.99. Additionally, an unrefined olive oil sample spiked with 1,2-PP-3-MCPD, 1,3-PP-2-MCPD and glycidyl palmitate (all standards TRC, Toronto, ON, Canada) with a total concentration of approximately 1 mg/kg each in their free forms, respectively, was used to control the completeness of the reaction. For measurement, the 7890A gas chromatograph coupled to 7000C triple quadrupole mass detector (Agilent Technologies, Santa Clara, CA, USA) was used, as well as HP-5 MS column 30 m × 0.25 mm × 0.25 µm (Agilent, Santa Clara, CA, USA). All the data were collected and processed using MassHunter Workstation Software, Quantitative Analysis for QQQ, Version B.08.00 (build 8.0.598.0), and Microsoft Excel 2007 software.

### 2.7. Statistics

The statistical processing of the results was performed using the OriginPro software (OriginPro 2018 SR1 b9.5.1.195). Differences between samples were assessed using a non-parametric Kruskal–Wallis test (H-test), followed by a Dunn test as a multiple comparison method. The null hypothesis was assumed to be valid if the estimated significance level was above 0.01–0.1. If the estimated significance level was between 0.01 and 0.1 test range, the null hypothesis was not definitely rejected. If a *p*-value ≥ 0.05, there was an assumption about low influence of the deodorisation degree (factor) on the studied value. And vice versa: if a *p*-value < 0.05, it was assumed that there was a tendency for the value to be influenced by the deodorisation degree. If the estimated significance level was below the 0.01–0.1 test range, the null hypothesis was rejected.

## 3. Results

To register differences caused by repetitive deodorisation, if any, the samples were analysed by means of measuring the fatty acid composition using GC-FID; measuring the peroxide value (PV) using titrimetry; measuring the anisidine value (AV) using spectrophotometry; and measuring the volatile organic compounds (VOCs), including oxidation products, using GC-FID/MS and MCPDE and GE using GC-MS/MS.

### 3.1. Change in Fatty Acids

**Figure 1:**
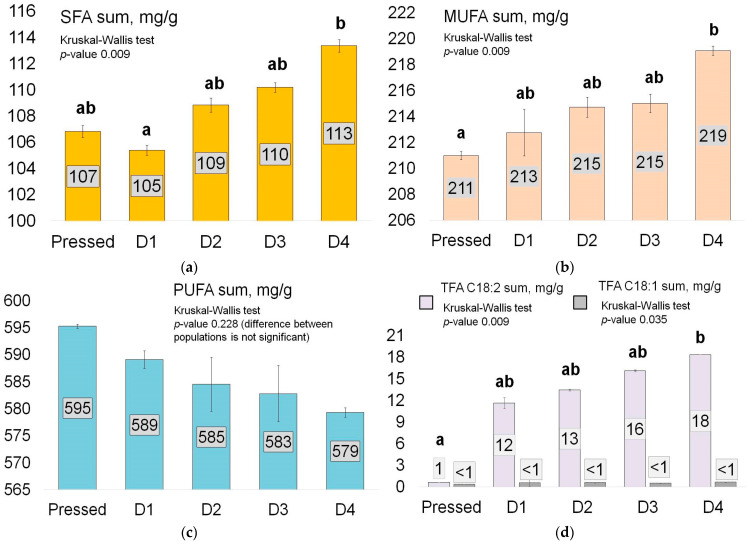
The changes in fatty acid content according to the degree of refining and deodorisation: (**a**) the sum of saturated FA, mg/g; (**b**) the sum of monounsaturated FA, mg/g; (**c**) the sum of polyunsaturated FA, mg/g; (**d**) the sum of trans-isomers FA, mg/g. The samples were analysed in duplicate, the values are mean, and error bars indicate standard deviations. Different letters above the bar charts indicate a statistically significant difference (*p* < 0.05).

Fatty acids represent the first compounds to undergo high-temperature and oxidative changes in edible fats and oils. Unless all the studied oils were at a very early stage of oxidation, slight differences in the content of FA groups depending on the degree of deodorisation were detected by the GC-FID method. The results are presented in Figure 1 in mg/g. The saturated FA (SFA) group consisted of myristic C14:0, palmitic C16:0, margaric C17:0, stearic C18:0, arachidic C20:0, behenic C22:0, and lignoceric C24:0 acids. The monounsaturated FA group (MUFA) consisted of palmitoleic C16:1 9-cis, oleic C18:1 9-cis, and gondoic C20:1 11-cis acids. The polyunsaturated FA group (PUFA) consisted of linoleic C18:2 and α-linolenic C18:3 acids. The trans-isomers FA group (TFA) contained elaidic C18:1 9-trans, isooctadecadienoic C18:2 9-trans,12-trans, 9-cis,12-trans-linoleic C18:2, and 9-trans,12-cis-linoleic C18:2 acids. The complete fatty acid profile, including mg/g and % data, can be found in the Supplementary Material (Table S1).

The content of SFA and MUFA exhibited a tendency to increase with the number of deodorisation cycles, with a difference between pressed and D4 samples of 5.6% and 3.8%, respectively. The content of SFA and MUFA in the D4 sample exhibited a statistically significant difference from those in the pressed sample at the *p* < 0.05 level. At the same time, the PUFA content exhibited a slight and insignificant decrease of 2.7% when comparing the pressed and D4 samples. Interestingly, there was a significant increase (at *p* < 0.05 level) in TFA content in the linoleic acid isomers (but not in the oleic acid isomers), namely, 9-cis-,12-trans-linoleic C18:2, 9-trans-,12-cis-linoleic C18:2, and C18:2 9-trans,12-trans iso-octadecadienoic. The difference between the pressed and D4 samples exceeded 18-fold, while that between the D1 and D4 samples was 1.5-fold. Previous studies have also identified a similar change in TFA following a single deodorisation cycle [28].

### 3.2. PV and AV Measurement

**Figure 2:**
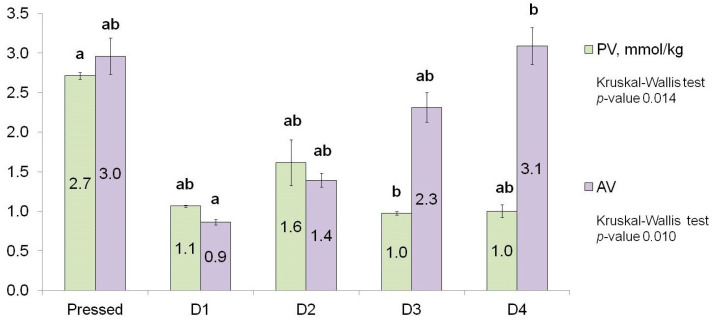
The peroxide and anisidine values in pressed sunflower oil and oils deodorised several times. The samples were analysed in duplicate (PV) and triplicate (AV). The values are mean; error bars indicate standard deviation. Different letters above the bar charts indicate a statistically significant difference (*p* < 0.05).

The peroxide value is one of the earliest indicators in the assessment of oil oxidation, which is used to measure the content of primary oxidation products, mostly peroxides [29]. At the same time, the anisidine value is usually used to detect lipid oxidation products in deeply oxidised, non-coloured oils that are free of any additives. This is due to the ability of *p*-anisidine to react with secondary oxidation products, mainly non-volatile mono- and diunsaturated aldehydes [30]. The analysis of peroxide and anisidine values in the studied oils revealed that the first deodorisation cycle lowered the content of compounds that can react with iodine and *p*-anisidine by 59.2% and 70%, respectively. However, these differences were not statistically significant (Figure 2).

The subsequent deodorisation cycles had a negligible impact on the amount of peroxides (the differences between pressed-D1, -D2, and -D4 samples were not significant). However, the content of compounds that reacted with *p*-anisidine exhibited a significant increase from the D1 to the D4 samples by 244%, reaching a value that was very close to the AV in the pressed oil.

### 3.3. Change in Volatile Profile

The volatile compounds in the aroma profile of edible oils and fats contain special group of substances, known as volatile oxidation products. The latter can serve as one of the marker and source of information during the initial stage of lipid oxidation [31]. In this study, HS-SPME followed by GC-FID/MS was used to detect and identify the analytes. The HS-SPME conditions were selected in a way that would prevent the formation of volatile oxidation products during the sample preparation. As it was mentioned in the Section 2.5, we focused only on identifying specific scent footprints and on comparative assessment of the volatile profiles between samples.

**Figure 3:**
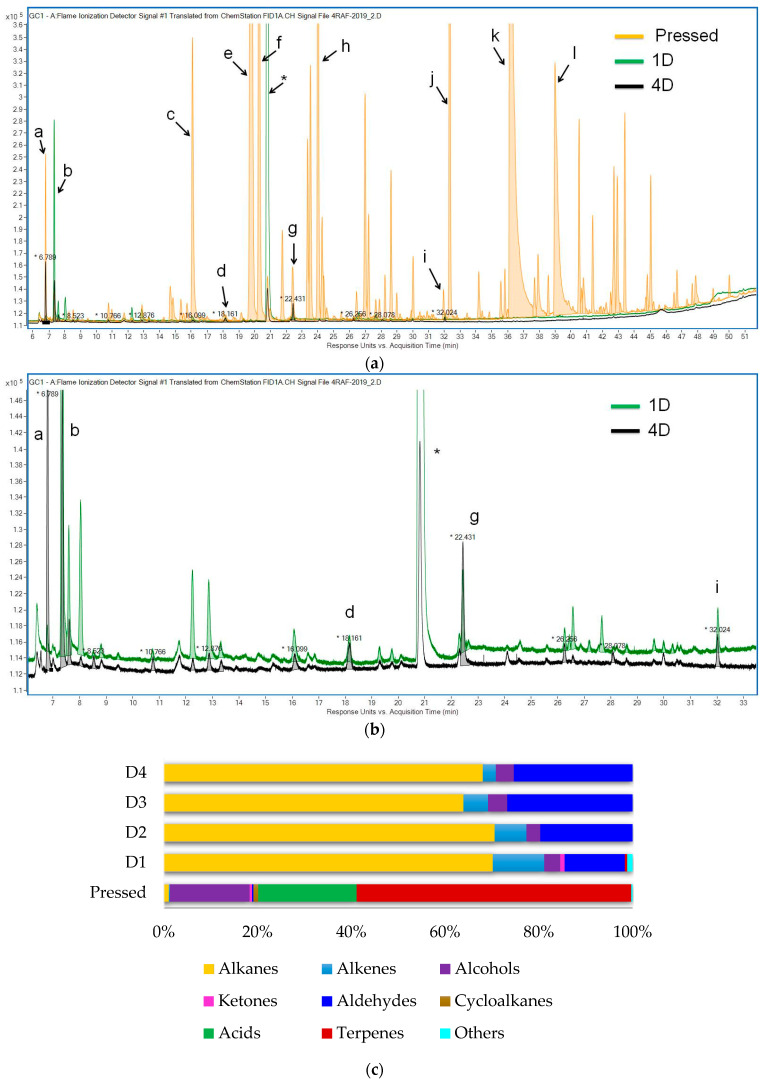
Volatile profile of sunflower oils: (**a**) pressed; (**b**) deodorised four times (D4); (**b**) the sum of polyunsaturated FA, mg/g; (**c**) normalised mass fractions of each substance group based on the sum of peak areas. In Figure 3a,b: a—Pentane, b—Hexane, c—Ethanol, d—Pentanal, e—α-Pinene, f—1-Propanol, g—Hexanal, h—Sabinene, i—(E)-2-Heptenal, j—1-Hexanol, k—Acetic acid, l—Propionic acid, *—Chloroform.

Figure 3a,b demonstrate the GC-FID chromatograms of the volatile compounds present in the headspaces of the SFO samples without processing and that have been deodorised once (D1) and four (D4) times. As it was expected, the volatile profiles were found to be completely different. The volatile profile of the pressed oil was abundant in terpene derivatives (α-pinene and sabinene), acids (acetic and propionic), and alcohols (ethanol, 1-propanol, and 1-hexanol). Refining and deodorisation had a great impact on the volatile profile of SFO: almost all terpenes and terpenoids, acids, and alcohols were missing. At the same time, alkanes (pentane and hexane) and aldehydes such as pentanal, hexanal, and (E)-2-hexenal increased.

Repeated deodorisation did not alter the volatile spectra of the refined SFO samples, but it induced an increase in the aldehyde content. This can be seen in the peak areas in Table 2.

Alkanes and alkenes were the only group consisting of the same compounds in pressed and refined SFOs. Pentane is considered to be an oleate and linoleate degradation product [32,33], and it was the main alkane in all samples, as expected. Heptane and octane, which are oleate degradation products, were also identified in these oils [32]. The source of hexane in the investigated samples is debatable because it could appear both naturally due to lipoxygenase action [34] and/or artificially if solvent extraction had taken place during oil processing. As for the alkenes, the first deodorisation caused the group to increase from 0.1% of the total peak areas in pressed SFO to 11.2% in D1 and then to decrease to 2.8% of the total peak areas in D4 (Figure 3c). The main alkenes in all samples were 1-octene and (Z)-2-octene, and the latter could arise from linoleic acid [32].

The aldehyde group was one of the few groups of volatiles that represent the aroma profile of processed sunflower oils. The most abundant substances here were pentanal, hexanal, and (E)-2-heptenal. Pressed oil had none of these: its characteristic aldehydes were 2-propenal, 2-methylbuthanal, and (E,E)-2,4-decadienal. The percentage of the aldehyde group in the total sum of volatiles was only 0.4%, which is much lower than 12.4%, 19.8%, 27.2%, and 25.3% in D1-D4 oils (Figure 3c). On the other hand, the absolute sum of aldehyde peak areas was much higher in the pressed oil than in the others. This is quite interesting when compared with the AV data. According to Figure 2, the content of compounds, mainly aldehydes, reacting with *p*-anisidine increased and reached the same value in pressed and D4 samples. The sum of the peak areas of the volatile aldehydes in the D4 sample did not reach the level in the pressed sample, which means that the AV may be mainly associated with non-volatile aldehydes than with volatile aldehydes.

Alcohols, acids, and monoterpenoids were the characteristic substances in the aroma profile of pressed SFO. The peak area of ethanol was several tens of times higher than that of processed SFOs, in agreement with [35]. The primary acids in pressed SFO were acetic acid (16.7%) and propionic acid (4.2%), which are produced during the processing of sunflower seeds [36]. Monoterpenoids were the largest volatile group and consisted of acyclic (β-myrcene), monocyclic, and bicyclic monoterpenes and a tricyclic sesquiterpene—calarene. A-pinene, sabinene, β-pinene, and three forms of verbenol represented 41.4%, 4.4%, 2.4%, 1.1%, 1.1%, and 1.0% of the total peak area, respectively, and had the most abundant peak areas in pressed SFO.

The presence of Maillard reaction products in pressed oil, namely, 2,5-dimethylpyrazine, 2-ethyl-5-methylpyrazine, and 2,3,5-trimethylpyrazine, should be noted. These substances are usually part of the typical volatiles of roasted products such as coffee [37]. It has been suggested that they could appear in sunflower seeds if the raw material has been subjected to high-temperature processing such as drying and then transferred to the pressed oil.

Thus, the first deodorisation partially or completely removed volatiles and lowered oxidation indices, but oxidation started again during the next deodorisation cycles, with new VOCs appearing and being released.

Overall, the list of identified substances was mainly in agreement with the previous results [38,39] except for 1-octen-3-ol, octanal, 3-octen-2-one, 2-octenal, nonanal, and 2-decenal, which were not found in the investigated oils.

### 3.4. MCPD Fatty Acid Esters and Glycidol Fatty Acid Esters Content

**Figure 4:**
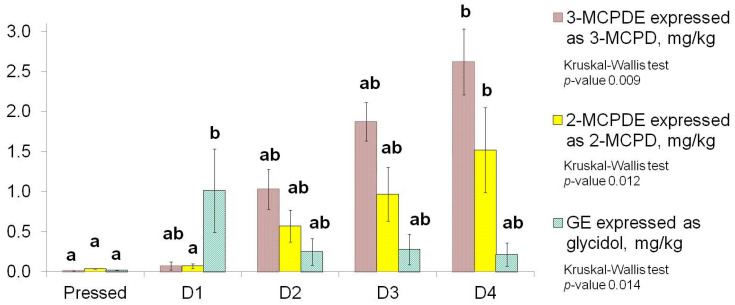
Change in 2-,3-MCPD fatty acid esters and glycidyl esters content, expressed as free 2-, 3-MCPD, and glycidol, respectively, depending on the number of deodorisation cycles. Samples were analysed in triplicate, values are mean, and error bars indicate standard deviation. Different letters above the bar charts indicate a significant difference (*p* < 0.05).

MCPD and glycidol were measured in all samples via long-term alkaline interesterification followed by purification, extraction, and GC-MS/MS determination. The results showed that the concentration of these analytes in the unrefined sunflower oil was relatively low and could not be measured reliably (<LOQ = 0.06 mg/kg), which is in agreement with previous data [40,41] (Figure 4).

The first refining and deodorisation caused a negligible change in MCPD content. The second deodorisation cycle led to an increase in the 3-MCPD content to 0.71 ± 0.31 mg/kg, the next one to 2.00 ± 0.27 mg/kg (D3), and the fourth to 2.81 ± 0.41 mg/kg (D4). Although these changes were not significant (i.e., pressed-D1, D1–D2, D2–D3, and D3–D4), the increase in the content of 3-MCPD between pressed-D4 and the next samples was.

The content of 2-MCPD changed in the same way as 3-MCPD, and its concentration was always about two times lower. The highest concentration was measured in the sample D4—1.47 ± 0.37 mg/kg, and this value was significantly different from the values in the pressed and D1 samples.

Conversely, the content of glycidyl esters, expressed as glycidol, increased significantly up to 0.73 ± 0.26 mg/kg as a result of the processing implemented for the oil and then decreased to 0.19 ± 0.09 mg/kg, although not significantly. The following deodorisation cycles did not cause any significant changes in the concentration of this compound.

All the tendencies detected are in agreement with the previous data, which stated that double refining is able to remove GEs, but MCPDEs are very stable during edible oil processing and, once formed, could hardly be removed [6].

The results of the statistical analysis using the Kruskal–Wallis test (H-test) are presented in Table 3 as quantile distributions of the values and estimated *p*-values. Only the results of PV, AV, MCPD, and glycidol content and fatty acids (excluding the content of TFA C18:1) were used in the analysis.

It can be seen that all *p*-values except for the *p*-value of the sum of polyunsaturated fatty acids were in the assigned range of 0.01–0.1. Therefore, the PUFA data were not used in the following Dunn’s test (see Table 4).

Table 4 shows that in the samples studied, values such as AV, 3-MCPD, 2-MCPD, glycidol, TFA C18:2, MUFA, and SFA were sensitive to the degree of refining and deodorisation and showed a reliable response at different stages of oil processing. In contrast, the PUFA content demonstrated statistically unreliable changes.

## 4. Discussion

The changes in TFA were found to be highly dependent on the content of C18:2 trans-isomers, which were carefully identified using the standard mixture. Although it was more expected to detect an increase in C18:3 TFAs because of the lower stability of α-linolenic acid compared to other unsaturated fatty acids, only a notable increase in C18:2 TFAs was found. At the same time, a very low amount of C18:1 9-trans elaidic acid was detected in pressed and deodorised oils (<1 mg/g), which did not have a significant impact on the total TFA content even after the fourth deodorisation. It is possible that the predominant formation of detectable amounts of C18:2 TFAs can be explained by the excess of linoleic acid in the fatty acid composition studied. This is evidenced by the fact that the percentage of linoleic acid in all samples was approximately 2.7 times higher than that of oleic acid and over 700 times higher than that of linolenic acid (see Supplementary Material Table S1).

Among the C18:2 TFAs, only C18:2 9-cis, 12-trans was detected in the pressed oil. The first refining and deodorisation process resulted in an increase in the C18:2 9-cis, 12-trans content and the formation of C18:2 9-trans, 12-cis and C18:2 9-trans, 12-trans. The ratio between C18:2 9-cis, 12-trans and C18:2 9-trans, 12-cis was 1.16 in the oil deodorised once and decreased to 1.10 in D4 oil due to the higher growth rate of C18:2 9-trans, 12-cis compared to C18:2 9-cis, 12-trans. These results are in agreement with the findings of authors in [42], who demonstrated that C18:2 9-trans, 12-cis exhibited a faster growth rate than C18:2 9-cis, 12-trans and that C18:2 9-trans, 12-trans was detected in quantifiable amounts after 180 min of heating the trilinolein at 180 °C.

The peroxide value in the studied samples was, as expected, higher in the pressed oil than in the refined oils. Although deodorisation was applied to obtain the samples D1-D4, their PVs were not close to zero as when measured immediately after processing (see data in Table 1). This discrepancy may be attributed to the fact that the samples (pressed, D1–D4) were frozen, delivered to the laboratory, and left to await analysis for up to two weeks, during which time they were stored at −22 °C. All deodorised samples had a relatively constant peroxide value, with the exception of sample D2, which confirms the ability of low temperatures to inhibit the oxidation process to a certain extent during the above-mentioned period. The same was found for olive oils, where PVs started to increase after 2–3 weeks of storage at −27 °C [43].

Conversely, the anisidine value exhibited a notable increase from samples D1 to D4, with the level in the D4 sample being equivalent to that observed in the pressed oil. It is noteworthy that the levels of volatile aldehydes, which are considered to be the main *p*-anisidine reactive species, exhibited a similar dynamic (see Table 2). However, the summed area of the D4 sample was found to be lower than that of the pressed oil, which may be attributed either to a lack of specificity in the anisidine value determination method or to the formation of non-volatile aldehydes during oil processing.

Our results on MCPDEs and GEs confirmed previous reports that unrefined oils do not contain these substances or contain them in trace amounts, except for palm oil, which is usually extracted under special conditions and contains elevated amounts of chlorinated substances and partial acylglycerides [44]. The latter are considered to be potential precursors for the formation of MCPDEs and GEs. Other pressed oils, including sunflower oil, may also contain these precursors, although in significantly less quantities [45]. Refining can also introduce at least chlorinated substances through the use of wash water [46] and bleaching clay [47]. It is considered that the formation of monochlorpropanediol esters requires partial acylglycerols, the chloride ion, and temperatures above 120 °C [41], while the formation of glycidol mostly depends on the presence of mono- and diacylglycerols and high-temperature heating (above 200 °C) [17].

In this study, D1-D4 oils were deodorised several times at 220 ± 2 °C for 75 ± 5 min, resulting in an increase in the MCPDEs content to 2.81 ± 0.41 mg/kg in the D4 sample. This level was more than two times higher than the maximum level permitted by the European Union for edible fats and oils, including sunflower, for release on the market or for use as an ingredient in foodstuffs, which is 1.25 mg/kg [48]. As for glycidyl esters, their content was close to the maximum level allowed for vegetable oils—1 mg/kg [48] after the first deodorisation. These data demonstrate the necessity of implementing mitigation strategies in any edible oil refining process, including both MCPDE formation prevention and removal steps [49].

The statistical processing of the results was able to detect alterations in the quality and safety of oils with the increase in the number of deodorising cycles, even at a minimal level of oxidation. However, it is important to note that the observed changes lacked a strict significance with *p* < 0.01. Nevertheless, it can be assumed that the more deodorisation cycles implemented for the same sunflower oil, the lower its quality and safety, and therefore its shelf life, at least in terms of increasing the content of peroxides and *p*-anisidine-reactive substances such as saturated and unsaturated aldehydes, as well as decreasing the content of polyunsaturated fatty acids. It is likely that these changes would have been more significant if a larger number of samples had been used.

In conclusion, the objective of this study was to demonstrate that each subsequent deodorisation cycle/intermediate processing may have a detectable impact on the quality and safety of unsaturated edible oils. This assumption was confirmed by the increase in C18:2 TFAs, AV, saturated and unsaturated volatile aldehydes, and 3-MCPDE content with the number of deodorisation cycles. This study did not aim to examine the shelf life of sunflower oils under the aforementioned conditions. It should also be noted that this study lacks a sufficient number of samples treated with subsequent deodorisation cycles to allow a precise evaluation of the differences revealed for statistical significance, as well as a dynamic examination of the natural antioxidants present in sunflower oils, such as vitamin E and sterols, which are also important micronutrients. These issues warrant further investigation.

In spite of this, this work can serve as a part of the evidence base to monitor and, if possible, limit the number of refining/deodorisation cycles for the same oil.

## 5. Conclusions

This first study was initiated to gain a better understanding of the effects of repeated deodorisation on sunflower oil and thus to ascertain whether an issue exists due to the official requirement of the intermediate processing step, which is deodorisation, following transportation in bulk, as stated in the FEDIOL documents [3,4] or this additional processing is a beneficial practice. For this purpose, pressed sunflower oil was repeatedly processed by means of deodorisation in order to investigate changes that may occur in the chemical composition, safety, and oxidative stability of the edible oil. To this end, measurements were made of the fatty acid composition, peroxide value, anisidine value, volatile profiles, and the levels of MCPDEs and GEs content.

The findings suggest that multiple deodorisations of the same oil, using the example of sunflower oil, may reduce its oxidative stability by lowering the content of polyunsaturated fatty acids and increasing the area of volatile aldehydes. The latter compounds, which were possibly derived from oleic, linoleic, and linolenic acids, were the most abundant oxidation products in processed sunflower oils. This group comprised acetaldehyde, pentanal, hexanal, (E)-2-hexenal, and (E)-2-heptenal, with hexanal being the main oxidation product. The total peak area of aldehydes increased progressively from the D1 to D4 samples. The alcohols; acids; and a lot of monoterpenoids, including α-pinene, sabinene, and β-pinene as major terpenoids, were mostly contained in pressed oil.

At the same time, the content of trans-fatty acids, unsaturated aldehydes, and monochloropropanediol fatty acid esters increased with the number of deodorisations. Such substances have potential adverse effects on human health.

The repeated deodorisation of refined single-component fats and oils, as well as more complex products such as spreads, shortenings, margarines, etc., may result in the excessive formation and contamination by MCPDEs without extra caution to prevent their formation. The possible preventive actions may include using new refining technologies or the modification of deodorisation conditions.

Therefore, one of the innovative results of this study is a base to revise the requirement of the intermediate processing step after transportation in bulk, which means at least one deodorisation step. Currently, this is a globally applicable standard for all edible fats and oils, including hard oils such as palm and liquid oils such as sunflower oils, which are to be transported in bulk by sea, road, and rail. Up to now, there are no officially approved documents to limit the total sum of processing steps as well as no scientific basis for amending the relevant legal norms.

Furthermore, this research provides a strong recommendation to limit the number of deodorising cycles implemented for the same oil. It also offers a foundation for additional studies with oils of various unsaturation degrees treated with a controlled number of deodorisation cycles in order to maintain their quality and safety from harvesting to the market.

## Figures and Tables

**Table 1:** Sunflower oil refining and deodorisation cycles.

	Pressed SFO	Refined and Deodorised SFO			
Sample code	Pressed	D1	D2	D3	D4
Number of refining cycles	0	1	1	1	1
Number of deodorisation cycles	0	1	2	3	4
PV, mmol/kg	3.5	0.3	1.1	0.2	0.5

**Table 2:** Volatile compounds in sunflower oils with different numbers of deodorisation cycles. I_K_—experimental Kovats indices, I_Kt_—theoretical Kovats indices, T’_R_—reduced retention time. Results presented as peak areas × 10^−7^ (average of two parallel FID measurements). Ol—oleic acid, L—linoleic acid, and Ln—linolenic acid.

Type of Oil						Pressed	D1	D2	D3	D4	Parent Fatty Acid
Compound Name	CAS	I_K_	I_Kt_	Aroma	T’_R_						
Alkanes (sum)						0.73	0.77	0.31	0.30	0.40	
Pentane	109-66-0	499	500		0.43	0.41	0.02	0.14	0.18	0.16	Ol/L
Hexane	110-54-3	601	600		0.99	0.22	0.74	0.16	0.11	0.23	
Heptane	142-82-5	696	700	sweet, ethereal	2.17	0.02	-	0.00 ^1^	0.00 ^1^	0.00 ^1^	Ol
Octane	111-65-9	800	800	gasoline	4.40	0.08	0.01	0.01	0.00 ^1^	0.00 ^1^	Ol
Alkenes (sum)						0.16	0.12	0.03	0.02	0.02	
1-Heptene ^2^	592-76-7	749	731-748		0.66	0.01	-	-	-	-	L
1-Octene	111-66-0	851	822-892	gasoline	5.99	-	0.03	0.01	0.00 ^1^	0.01	
(Z)-2-Octene	7642-04-8	868	862-882		6.51	0.12	0.07	0.02	0.01	0.01	L
(E)-2-Octene ^2^	13389-42-9	880	852-867		6.97	0.03	0.01	0.01	0.01	0.00	
Alcohols (sum)						11.56	0.04	0.01	0.02	0.02	
Ethanol	64-17-5	939	883-972		9.73	1.63	0.04	0.01	0.02	0.02	
1-Propanol	71-23-8	1064	988-1086	alcoholic, fermented, fusel, mouldy	13.92	3.75	-	-	-	-	
2-Methylpropanol	78-83-1	1119	1043-1129	ethereal, wine, bark	16.05	0.35					
2-Propen-1-ol	107-18-6	1141	1097-1167	spicy, mustard	16.99	0.39					
2-Methylbutanol	137-32-6	1220	1158-1244	fried, wine, onion, fruit, fusel, alcohol, whiskey	20.62	1.09					
1-Pentanol	71-41-0	1265	1200-1294	fusel, oils, sweet	22.26	0.62					L
1-Hexanol ^2^	111-27-3	1368	1308-1349	essential, fusel, oily, fruity, alcoholic, sweet, freshness	25.98	3.16					
1-Heptanol	111-70-6	1419	1405-1484	musty, leafy, violet, herbal, green, sweet, woody, peony	29.46	0.19					
Ketones (sum)						0.36	0.01				
2-Butanone	78-93-3	915	866-950	acetone, essential, fruity, camphor	8.48	0.24					
2-Pentanone	107-87-9	971	938-1015	sweet, fruity, essential, wine, banana, woody	11.68	0.07					
2-Heptanone	110-43-0	1205	1145-1214	fruity, spicy, sweet, herbal, coconut, woody	20.08		0.01				
3-Octanone	106-68-3	1280	1205-1300	fresh, herbal, lavender, sweet, mushroom	22.84	0.04					
2-Octanone	111-13-7	1310	1262-1310	earthy, natural, woody, herbal	23.97	0.01	0.00 ^1^				
Aldehydes (sum)						0.23		0.09	0.13	0.15	
incl. α,β-unsaturated						0.03					
Acetaldehyde	4124-63-4	714	689-744	cabbage	2.45		0.01	0.01	0.01	0.00 ^1^	L/Ln
Propanal	123-38-6	808	747-828	earthy, alcoholic, wine, whiskey, cocoa, nutty	4.62	0.04					L/Ln
2-Methylbutanal	96-17-3	925	880-963	musty, cocoa, coffee, nutty	8.99	0.13					
Pentanal	110-62-3	998	929-1013	fermented, bakery, fruit, nutty, berry	11.80		0.01	0.02	0.02	0.03	L/Ln
Hexanal	66-25-1	1120	1034-1127	herbal, oily	16.07		0.06	0.04	0.08	0.09	L
Heptanal	111-71-7	1209	1148-1219	buttery, citrus, rancid	20.22		0.03				L/Ln
(E)-2-Hexenal ^2^	6728-26-3	1251	1184-1236	sweet, almond, fruity, fresh, leaves, apple, plum, vegetable	21.73				0.01	0.01	Ln
(E)-2-Heptenal	18829-55-5	1359	1273-1366	freshness, fatty	25.67		0.02	0.01	0.02	0.02	Ol/L
(E,E)-2,4-Decadienal ^2^	2363-88-4	1859	1768-1858	orange, sweet, fresh, citrus, greasy, freshness	40.97	0.03					Ol/L
Cyclic (sum)						0.49					
Methylcyclopentane ^2^	96-37-7	681	675		1.91	0.04					
Cyclohexane	110-82-7	734	712-766		2.80	0.01					
3-Methylfuran	930-27-8	889	832-901		7.33	0.01					
2-Pentylfuran	3777-69-3	1255	1193-1265	fruity, green, earthy, nutty, vegetable, metallic	21.89	0.18					
2-Furanmethanol	98-00-0	1687	1613-1698	alcoholic, chemical, musty, sweet, caramel, bread, coffee	36.15	0.09					
Benzyl alcohol ^2^	100-51-6	1920	1821-1919	floral, rose, phenolic, balsamic	42.62	0.06					
Benzene ethanol ^2^	60-12-8	1959	1856-1956	floral, rose, dried rose, floral, rose water	43.65	0.10					
Acids (sum)						14.60					
Acetic	64-19-7	1481	1400-1498	sharp, acrid, sour, acetic	29.77	11.16					
Propionic	79-09-4	1571	1474-1486	spicy, sour, cheesy, acetic	32.63	3.27					
Hexanoic	142-62-1	1879	1797-1885	sour, fatty, cheesy	41.52	0.17					
Terpene derivatives (sum)						40.81					
Tricyclene	508-32-7	1031	993-1047		12.80	0.04					
α-Pinene	80-56-8	1050	989-1077	fresh, camphor, sweet, pine, earthy, woody	13.43	28.50					
Terpene derivative 1	471-84-1	1095			15.04	0.04					
Camphene	79-92-5	1104	1033-1115	woody, herbal, fir, camphor, terpene	15.24	0.44					
β-Pinene	127-91-3	1145	1065-1158	dry, woody, fresh, pine, hay, freshness, resinous	17.17	1.62					
Sabinene	3387-41-5	1155	1074-1156	woody, terpene, citrus, pine, spices	17.65	3.05					
Terpene derivativee 2	36262-09-6	1160			17.91	0.49					
Verbenene	4080-46-0	1162			18.00	0.19					
β-Myrcene	123-35-3	1185	1113-1192	pepper, terpene, pungent, balsamic, plastic	19.12	0.02					
Terpene derivative 3		1205			20.09	0.12					
Limonene	138-86-3	1226	1152-1245	citrus, herbal, terpene, camphor	20.84	0.46	0.01				
Eucalyptol	470-82-6	1239	1167-1253	eucalyptus, herbal, camphor, medicinal	21.30	0.11					
γ-Terpinene	99-85-4	1274	1200-1293	oily, woody, terpene, lemon, lime, tropical herbs	22.63	0.11					
Kumene	99-87-6	1300	1232-1322	fresh, citrus, terpene, woody, special	23.65	0.28					
Terpene derivative 4		1313			24.09	0.05					
α-Pinene epoxyde ^2^	1686-14-2	1421	1345-1384	freshness	27.81	0.21					
Terpene derivative		1464			29.21	0.17					
Camfolenal	4501-58-0	1537	1439-1793	herbal, freshness, woody, amber, leafy	31.56	0.43					
Verbenol 1		1557			32.20	0.17					
Verbenol 2		1620		balsamic	34.15	0.77					
Kalarene ^2^	17334-55-3	1649	1544		35.01	0.41					
Verbenol 3		1693		fresh, pine, ozone	36.36	0.70					
Pinocarveol ^2^	5947-36-4	1700	1632-1690	camphor, woody-pine, balsamic	36.56	0.67					
Verbenol 4		1717		balsamic	37.04	0.75					
Terpene derivative 5		1768			38.46	0.15					
Verbenon ^2^		1775	1676-1742	camphor, menthol, celery	38.68	0.56					
Terpene derivative 6		1828			40.15	0.06					
Mirtenol ^2^		1835	1747-1831	camphor, menthol, celery	40.34	0.15					
Trans-carveol	1197-07-5	1871	1801-1884	mint, solvent, cumin	41.31	0.08					
Others (sum)						0.20	0.01				
Ethyl acetate	103-45-7	902	856-917	floral, pink, sweet, honey, fruity, tropical	7.91	0.02	0.01				
2,5-Dimethylpyrazine	123-32-0	1357	1274-1358	cocoa, roasted nuts, roast beef, woody, herbs	25.58	0.12					
2-Ethyl-5-methylpyrazine	13360-64-0	1428	1341-1432	coffee beans, nutty, herbal, roasted, earthy, powdery, cocoa, baked potatoes	28.03	0.03					
2,3,5-Trimethylpyrazine ^2^	14667-55-1	1442	1341-1432	nut peel, earthy, powdery, cocoa, baked potatoes, roasted peanuts, hazelnuts, musty	28.48	0.03					

^1^ Compound’s area <0.005 × 10^−7^. ^2^ Theoretical Kovats indices do not match experimental Kovats indices, but matching criteria with NIST library spectra is still >700. This may be associated with poor Kovats indices data for polar columns.

**Table 3:** Quantile distribution of the values as results of the Kruskal–Wallis test (H-test). Me—mean.

		Pressed			D1			D2			D3			D4		
Quantiles	*p*-Value	Q1	Me	Q3	Q1	Me	Q3	Q1	Me	Q3	Q1	Me	Q3	Q1	Me	Q3
PV	0.014	2.7	2.7	2.7	1.1	1.1	1.1	1.5	1.6	1.7	1.0	1.0	1.0	1.0	1.0	1.0
AV	0.010	3.0	3.0	3.0	0.9	0.9	0.9	1.4	1.4	1.4	2.3	2.3	2.4	3.0	3.0	3.2
3-MCPD	0.009	0.02	0.02	0.03	0.06	0.07	0.07	0.84	0.85	0.89	2.19	2.20	2.22	3.01	3.14	3.19
2-MCPD	0.012	0.00	0.00	0.00	0.00	0.00	0.00	0.29	0.29	0.34	0.98	0.99	1.00	1.46	1.49	1.49
Glycidol	0.014	0.01	0.01	0.02	0.63	0.63	0.64	0.14	0.15	0.16	0.20	0.20	0.21	0.15	0.16	0.18
TFA C18:2	0.009	1	1	1	11	12	12	13	14	14	16	16	16	18	18	18
PUFA	**0.228**	595	595	595	589	589	590	583	585	586	581	583	584	579	580	580
MUFA	0.009	211	211	211	212	213	213	214	215	215	215	216	216	219	219	219
SFA	0.009	107	107	107	105	106	106	108	109	109	110	110	110	113	114	114

**Table 4:** The obtained Dunn’s test *p*-values related to the influence of the degree of deodorisation on some sunflower oil values and parameters studied. “Pr” is pressed oil. *p*-values in bold are between 0.01 and 0.1 range; orange means that the *p*-value is in a range of 0.01–0.05; green is *p*-value in a range of 0.05–0.1.

	PV	AV	3-MCPD	2-MCPD	Glycidol	TFA C18:2	MUFA	SFA
Pr-D1	0.158	**0.063**	0.457	1.000	**0.030**	0.457	0.457	0.457
Pr-D2	0.457	0.158	0.176	0.285	0.372	0.176	0.176	0.457
Pr-D3	**0.039**	0.413	**0.069**	0.109	**0.085**	**0.069**	**0.069**	0.176
Pr-D4	**0.057**	0.558	**0.030**	**0.045**	0.176	**0.030**	**0.030**	**0.069**
D1-D2	0.413	0.457	0.457	0.285	**0.085**	0.457	0.457	0.176
D1-D3	0.270	0.176	0.176	0.109	0.372	0.176	0.176	**0.069**
D1-D4	0.413	**0.033**	**0.069**	**0.045**	0.176	**0.069**	**0.069**	**0.030**
D2-D3	**0.094**	0.457	0.457	0.457	0.270	0.457	0.457	0.457
D2-D4	0.142	**0.077**	0.176	0.176	0.558	0.176	0.176	0.176
D3-D4	0.733	0.196	0.457	0.457	0.558	0.457	0.457	0.457

## Data Availability

The original contributions presented in the study are included in the article/Supplementary Material, further inquiries can be directed to the corresponding author.

## Supplementary Materials

The following supporting information can be downloaded at: https://www.mdpi.com/article/10.3390/foods13162555/s1, Table S1: Fatty acid composition of sunflower oils after different numbers of deodorisation cycles.
